# Virtual non-calcium dual-energy CT: clinical applications

**DOI:** 10.1186/s41747-021-00228-y

**Published:** 2021-09-03

**Authors:** Tommaso D’Angelo, Moritz H. Albrecht, Danilo Caudo, Silvio Mazziotti, Thomas J. Vogl, Julian L. Wichmann, Simon Martin, Ibrahim Yel, Giorgio Ascenti, Vitali Koch, Giuseppe Cicero, Alfredo Blandino, Christian Booz

**Affiliations:** 1 Department of Biomedical Sciences and Morphological and Functional Imaging, University Hospital Messina, Messina, Italy; 2 Division of Experimental Imaging, Department of Diagnostic and Interventional Radiology, University Hospital Frankfurt, Theodor-Stern-Kai 7, 60590 Frankfurt am Main, Germany

**Keywords:** Algorithms, Bone marrow, Calcium, Edema, Tomography (x-ray, computed)

## Abstract

Dual-energy CT (DECT) has emerged into clinical routine as an imaging technique with unique postprocessing utilities that improve the evaluation of different body areas. The virtual non-calcium (VNCa) reconstruction algorithm has shown beneficial effects on the depiction of bone marrow pathologies such as bone marrow edema. Its main advantage is the ability to substantially increase the image contrast of structures that are usually covered with calcium mineral, such as calcified vessels or bone marrow, and to depict a large number of traumatic, inflammatory, infiltrative, and degenerative disorders affecting either the spine or the appendicular skeleton. Therefore, VNCa imaging represents another step forward for DECT to image conditions and disorders that usually require the use of more expensive and time-consuming techniques such as magnetic resonance imaging, positron emission tomography/CT, or bone scintigraphy. The aim of this review article is to explain the technical background of VNCa imaging, showcase its applicability in the different body regions, and provide an updated outlook on the clinical impact of this technique, which goes beyond the sole improvement in image quality.

## Key points

- Virtual non-calcium (VNCa) computed tomography (CT) imaging provides new relevant clinical information compared to standard CT.
- VNCa improves CT sensitivity and specificity to assess bone marrow disorders.
- VNCa CT may serve as an alternative to magnetic resonance imaging in case of contraindications.

## Background

Since the advent of dual-energy computed tomography CT (DECT), numerous and noteworthy advantages over conventional CT have been investigated such as image optimization, artifact reduction, and the ability to provide additional information regarding tissue composition [1–8].

Among different DECT applications, virtual non-calcium (VNCa) has become an increasingly popular technique due to its ability to subtract calcium from anatomical structures, resulting in better assessment of numerous pathological conditions that might be masked on standard CT. Firstly introduced in 2009, VNCa has found its mainstream applications in removing calcified plaques from vessels and depicting cancellous bone and bone marrow changes [9–13].

Bone marrow edema (BME) or “bone bruise,” is considered the biomarker of injury of the skeletal system and is associated with a reduction of fat component in the trabecular bone, replaced by edema and hemorrhage. Magnetic resonance imaging (MRI) represents the reference standard technique for the assessment of bone marrow disorders [14]. DECT may be considered a potentially cheaper, faster, and comprehensive imaging alternative through the creation of VNCa reconstructions, and it is aiming to provide as detailed information as MRI for a number of clinical indications [15].

A large body of evidence has shown the potential of DECT for collagen-based tendon and ligament imaging or to differentiate hyperdense lesions from calcium, such as urate crystals in patients with gout [16–20]. In this setting, DECT’s ability to provide information regarding an additional imaging parameter such as BME derived from VNCa images might be particularly helpful for a multiparametric approach to inflammatory, infiltrative, and degenerative disorders as well as in an emergency setting for traumatized patients.

This article discusses and summarizes the current clinical applications of VNCa imaging including different DECT platforms, their basic principles of physics, and areas of potential development.

## Basic principles of DECT

While standard single-energy CT uses a single polychromatic x-ray beam with a single peak energy of 120 kV, DECT systems allow for simultaneous image acquisition at two different voltages.

At the energy levels used in clinical routine, attenuation of biological tissues is dependent on two major interactions: Compton scattering and photoelectric effects. Compton scattering is proportional to electron density and has little energy dependence. On the other hand, the photoelectric effect is dependent on the energy of the x-ray beam (*E*), as well as on the atomic number (*Z*) of the element, and it is predominant at lower energies and near the element K-edge.

On single-energy CT platforms, the measurement of attenuation using a single x-ray spectrum does not allow for the differentiation of materials, because the attenuation coefficient is not unique. In particular, it depends either on the energy of the x-ray beam or on the concentration of a material. This means that at a given energy, a lower concentration of a higher *Z* material (*i.e*., iodine) may have the same attenuation as the higher concentration of a lower *Z* material (*i.e*., calcium). However, since materials have unique attenuation profiles at different energy levels, DECT aims to identify different elements exploiting mathematical algorithms based on their linear attenuation coefficients in order to achieve the so-called *material decomposition* [21–23].

DECT’s ability to discriminate between different materials is highly proportional to the dual-energy ratio (DE_ratio_). Since DECT uses two different energies for measuring attenuation, DE_ratio_ is defined as the ratio of the attenuation of a given material on a low-kV dataset to the attenuation of the same material on a high-kV dataset. The energy-dependent attenuation differences of elements within a voxel allow DECT to exploit two- and three-material decomposition. The ability to differentiate the DE_ratio_ of two materials also depends on the separation between low- and high-energy spectra and the *Z* of the evaluated materials.

However, DECT algorithms work better when materials have high atomic numbers, because these are characterized by large differences in attenuation at different photon energies (*i.e*., iodine and calcium). At tube voltages used in clinical routine, it is possible to generate virtual unenhanced images by subtraction of iodine from contrast-enhanced DECT examinations or VNCa series by subtraction of calcium [5, 15, 20, 24, 25].

Different vendors offer diverse DECT technologies:

1. *Dual-source CT (DSCT) platforms*, consisting of two x-ray tubes that generate two beams at different voltages installed at about 90° from each other, which have the advantage of high temporal resolution; although two x-ray sources are involved, the radiation dose administered to the patient is usually divided between the two, resulting in dose neutrality of second- and third-generation DSCT scanners compared with conventional CT [25, 26];
2. *Single-source with sequential acquisition CT platforms*, which consist of two scans acquired consecutively at different tube potentials followed by co-registration for post-processing, offering the advantage of a full field of view and the drawback of poor temporal resolution as the patient is scanned twice, with an increase of radiation dose;
3. *Single-source twin-beam platforms*, in which a two-material filter splits the x-ray beam into high- and low-energy spectra on the *z*-axis before it reaches the patient;
4. *Single-source tube voltage switching CT platforms*, in which the x-ray tube alternates high and low potentials several times within the same rotation, which have the advantage of a full field of view;
5. *Dual-layer detector CT platforms*, which involve a superficial and a deep layer within the same detector plate that simultaneously collect low-energy data by the superficial layer, and high-energy data by the deep layer.

However, most of the current experience with VNCa algorithms published in scientific literature has been performed on second- and third-generation DSCT platforms (Tables 1, 2, 3, and 4), which may be explained with wider availability of this particular DECT technology compared to the other ones.

**Table 1:** Virtual non-calcium computed tomography (CT) ability to differentiate hemorrhage from parenchymal calcifications on dual-energy head scans

Authors, year [reference]	DECT platform	Study type	Qualitative analysis (sensitivity, specificity, accuracy)	Quantitative analysis cutoff (sensitivity, specificity, accuracy)	Reference standard
Wiggins et al., 2019 [19]	DSCT	Clinical (137 patients)	100%, 100%, 100%	44 HU (100%, 93%, 95%)	MRI
Hu et al., 2016 [17]	DSCT	Clinical (62 patients)	96%, 100%, 99%	NA	MRI or clinical follow-up
Nute et al., 2015 [18]	VSCT	Phantom	NA	50 HU (NA, NA, 90%)	MRI

*DECT* Dual-energy computed tomography, *DSCT*, Dual-source CT, *HU* Hounsfield units, *MRI* Magnetic resonance imaging, *NA* Not available, *VSCT* Voltage-switching CT

**Table 2:** Virtual non-calcium computed tomography (CT) ability to depict traumatic bone marrow edema on dual-energy spine scans

Authors, year [reference]	DECT platform	Number of patients	Qualitative analysis (sensitivity, specificity, accuracy)	Quantitative analysis cutoff (sensitivity, specificity, accuracy)	Reference standard	Spine site
Wang et al., 2020 [51]	DSCT	20	85%, 97%, 93%	-12 HU (95%, 86%, 98%)	MRI	T/L
Jeong et al., 2020 [52]	DSCT	31	83%, 99%, 99%	NA	MRI	T/L
Booz et al., 2020 [29]	DSCT	52	93%, 95%, 90%	-43 HU (85%, 95%, 97%)	MRI	S
Foti et al., 2019 [58]	DSCT	76	88%, 92%, 90%	cutoff NA (92%, 90%, 91%)	MRI	T/L
Diekhoff et al., 2019 [53]	SACT	70	72%, 70%, NA	NA	MRI	T/L
Frellessen et al., 2018 [45]	DSCT	51	96%, 96%, 98%	NA	MRI	T/L
Diekhoff et al., 2017 [57]	SACT	9	88%, 100%, NA	NA	MRI	T/L
Petristsch et al., 2017 [54]	DSCT	22	64%, 99%, 93%	-47 HU (92%, 82%, 84%)	MRI	L
Kaup et al., 2016 [55]	DSCT	49	90%, 90%, 95%	NA	MRI	T/L
Bierry et al., 2014 [10]	DSCT	20	84%, 97%, 95%	cutoff NA (85%, 82%, NA)	MRI	T/L
Wang et al., 201 3[27]	DSCT	63	NA	-80 HU (96%, 98%, 97%)	MRI	T/L

*DECT* Dual-energy computed tomography, *DSCT* Dual-source computed tomography, *SACT* Sequential acquisition computed tomography, *HU* Hounsfield units, *NA* Not available, *MRI* Magnetic resonance imaging, *T* Thoracic, *L* Lumbar, *S* Sacral

**Table 3:** Virtual non-calcium computed tomography potential to visualize non-traumatic bone marrow edema on dual-energy scans of spine and pelvic girdle

Authors, year [reference]	DECT platform	Number of patients	Qualitative analysis (sensitivity, specificity, accuracy)	Quantitative analysis cutoff (sensitivity, specificity, accuracy)	Reference standard	Disorder	Anatomical site
Shinohara et al., 2020 [61]	DSCT	53	NA	NA	MRI	Disc degeneration	Spine
Foti et al., 2020 [74]	DSCT	59	95%, 86%, 93%	NA	MRI	Sacroiliitis	Hip
Chen et al., 2020 [71]	DSCT	40	81%, 94%, NA	-44 HU (76%, 91%, NA)	MRI	Sacroiliitis	Hip
Gruggeberger et al., 2020 [73]	DSCT	47	93%, 94%, NA	-35 HU (94%, 83%, NA)	MRI	Sacroiliitis	Hip
Abdullayev et al., 2019 [56]	DLCT	21	85%, 84%, 84%	NA	MRI	Vertebral metastases	Spine
Booz et al., 2019 [34]	DSCT	41	91%, 92%, 94%	NA	MRI	Disc herniation	Spine
Wu et al., 2019 [70]	DSCT	47	93%, 94%, 92%	-33 HU (90%, 83%, 83%)	MRI	Sacroiliitis Spondylarthritis	Spine/Hip
Kosmala et al., 2018 [12]	DSCT	53	NA	-36 HU (100%, 97%, 99%)	MRI	Multiple myeloma	Spine
Kosmala et al., 2018 [66]	DSCT	34	91%, 91%, 91%	-45 HU (93%, 92%, 93%)	MRI	Multiple Myeloma	Spine

*DECT* Dual-energy computed tomography, *DLCT* Dual-layer computed tomography, *DSCT* Dual-source computed tomography, *HU* Hounsfield units, *NA* Not available, *MRI* Magnetic resonance imaging

**Table 4:** Virtual non-calcium computed tomography ability to depict traumatic bone marrow edema on dual-energy scans of appendicular skeleton

Author	DECT platform	Number of patients	Qualitative analysis (sensitivity, specificity, accuracy)	Quantitative analysis cutoff (sensitivity, specificity, accuracy)	Reference standard	Anatomical site
Yang et al., 2020 [81]	DSCT	156	92%, 93%, 93%	NA	MRI	Knee
Booz et al., 2020 [82]	DSCT	56	96%, 97%, 97%	-51 HU* (96%, 97%, 96%)	MRI	Knee
Yadav et al., 2020 [83]	DSCT	40	94%, 91%, 92%	NA	MRI	Lower limb
Wang et al, 2019 [80]	DSCT	35	88%, 98%, 95%	-67 HU (81%, 99%, 90%)	MRI	Knee
Booz et al., 2019 [85]	DSCT	62	92%, 97%, 98%	-53 HU (82%, 95%, 98%)	MRI	Calcaneus
Foti et al., 2019 [87]	DSCT	40	92%, 86%, 90%	-20 HU (88%, 92%, 87%)	MRI	Ankle
Jang et al., 2019 [36]	DSCT	35	100%, 100%, 100%	-55 HU (100%, 94%, 95%)	Standard CT	Hip
Ali et al., 2018 [86]	DSCT	24	NA	6 HU (100%, 99%, 100%)	Visual assessment	Wrist
Kellock et al., 2017 [37]	DSCT	118	100%, 100%, NA	NA	Clinical follow-up	Hip
Reddy et al., 2015 [69]	DSCT	25	90%, 40%, NA	NA	Clinical follow-up	Hip
Guggenberger et al., 2012 [47]	DSCT	30	90%, 81%, 97%	NA	MRI	Ankle

*BME* Bone marrow edema, *CT* Computed tomography, *DECT* Dual-energy computed tomography, *DSCT* Dual-source computed tomography, *HU* Hounsfield units; *NA* Not available, *MRI* Magnetic resonance imaging. *Cutoff value refers to tibial BME

## Technical background of VNCa CT imaging

Due to three-material decomposition, the VNCa algorithm estimates the amount of calcium on the DECT dataset and subtracts it from images to highlight the anatomical structures that can be covered with bone mineral or gross calcifications.

On VNCa imaging, the bone marrow attenuation represents CT values of yellow and red marrow. In particular, using the characteristic slope of the DE_ratio_ of calcium, bone mineral voxels are projected to the CT value of water (0 HU for both 100 kV and Sn140 kV) [27, 28]. As a consequence, the differences among voxels reflect mainly the water and fat content in the bone marrow. These differences can be visually interpreted, using color-coded maps, or quantitatively assessed by means of regions of interest and expressed in HU [22, 29–31].

However, most of the cutoff values that have been suggested to differentiate BME from normal bone marrow are still quite heterogeneous, and range between -80 and 6 HU. These differences might depend either on the anatomical region evaluated or on the type of DECT platform used.

Image quality on VNCa datasets is also influenced by DECT scanning parameters. The use of higher spectral separation allows for a precise assessment of bone mineral content [32]. Best results have been obtained with a DE_ratio_ of 70/150 kV. However, when a wide DE_ratio_ may not be recommended because of the increase in image noise, such as scanning the abdomen and pelvis, higher radiation doses help provide optimal image quality [31]. Pitch and rotation time do not considerably affect image quality, although spiral artifacts can appear when the pitch is too low.

Color-coded VNCa datasets are usually automatically processed from raw data of most modern DECT platforms, with processing time lasting few minutes, and showing the potential to be used in routine clinical practice [33, 34]. Slice thickness of 1–2 mm and smoother reconstruction kernels are recommended and datasets should be reformatted along two anatomical planes for optimal qualitative evaluation [35].

Technical limitations of VNCa imaging should also be taken into account. In fact, it has been demonstrated the inability to accurately visualize minor alterations in marrow attenuation directly adjacent to cortical bone due to incomplete masking of the cortex and to spatial averaging. Incomplete subtraction of cortical or cancellous bone might also occur in the case of arthrosis, and in the presence of gas or severe osteosclerosis, which causes beam hardening artifacts that may result in a lack of visible edema even in the presence of a fracture. For this reason, any potential user of VNCa imaging should be aware of its potential pitfalls [36, 37].

## VNCa clinical applications

### Head

Intracranial calcifications are common findings on head CT scans [38]. They are often caused by dystrophic processes within the choroid plexus and basal ganglia or might occur as part of different disorders, such as tuberous sclerosis, cysticercosis, Sturge-Weber syndrome, and slowly growing brain tumors (*i.e*., meningiomas, dermoids, craniopharyngiomas, and oligodendrogliomas) [39].

In an emergency setting, any focal source of intracranial hyperattenuation might confound diagnosis, especially if intracranial hemorrhage needs to be excluded. In a conventional CT scan of the head, any lesion with attenuation levels greater than 100 HU is classified as a calcification [40]. However, fresh blood usually has a density of 50–65 HU whereas attenuation of calcifications can vary between 70 and 200 HU [41]. Hence, this standard criterion may fail for lesions with attenuation levels inferior to 100 HU, where values tend to overlap.

Currently, the proposed imaging techniques for differentiating hemorrhage from calcification include MRI-based techniques such as quantitative susceptibility mapping and gradient-echo imaging [40].

**Fig. 1:**
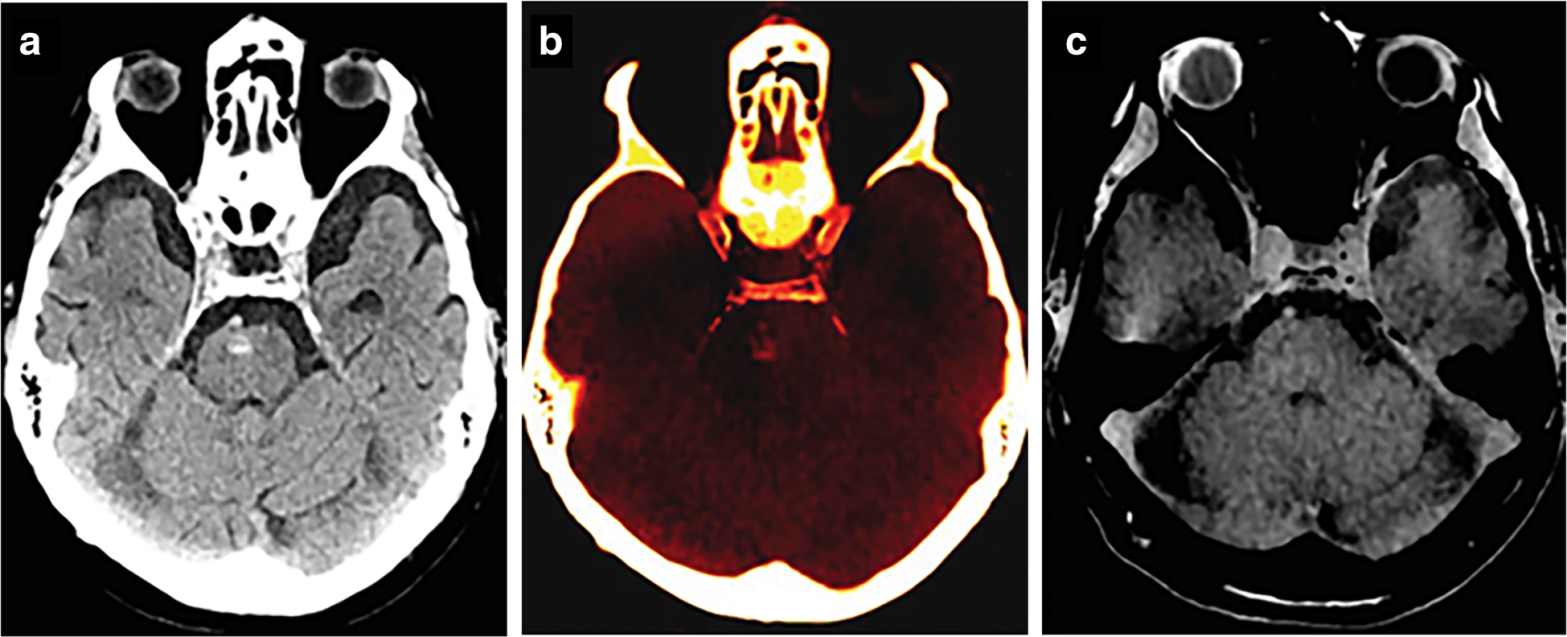
Head dual-energy computed tomography scan shows a focal hyperattenuation in the pons classified as indeterminate at standard grayscale series (**a**). This 80-year-old patient with a history of hypertension presented to the emergency department with altered mental status. On the calcium-overlay image (**b**), the hyperattenuation focus remains visible, while it is not apparent on the virtual non-calcium image (**c**), suggestive for parenchymal calcification. The finding was confirmed by magnetic resonance imaging. From Hu et al. [17]

VNCa has been shown to accurately differentiate between calcification and hemorrhage, even in lesions with attenuation comprised between 50 and 100 HU (Fig. 1) [18]. In clinical studies, VNCa with a cutoff of 44 HU has shown high diagnostic performance for differentiation of small foci of intracranial hemorrhage from calcium (Table 1) [17, 19].

### Spine

#### Fractures

MRI and CT are currently considered the diagnostic imaging modalities of choice to evaluate spine disorders [42]. CT imaging is indicated in emergency trauma settings to detect hypodense fracture lines due to its excellent spatial resolution [42, 43]. On the other hand, MRI is the reference standard technique for the evaluation of nerves, musculotendinous structures, and bone marrow disorders. Moreover, MRI is particularly useful to diagnose BME secondary to trauma, which allows establishing the chronicity of a fracture by the presence of interstitial fluid [44–46]. However, MRI access can be limited in routine trauma settings due to its high costs and long acquisition times, which require prolonged and potentially painful patient positioning [32, 46–48].

While single-energy CT is not able to remove bone trabeculations and to uncover subtle bone marrow attenuation changes, DECT allows for BME assessment [20, 49–55]. Moreover, its rapid acquisition time lends it favorably to emergency settings [46]. Several studies have been carried out to evaluate the diagnostic performance of VNCa reconstructions to detect acute vertebral fractures [56–58]. BME detection has been qualitatively assessed by using color-coded images and quantitatively using region-of-interest-based measurements of bone marrow attenuation. When microfractures are present within the cancellous bone, bone marrow attenuation increases since its fatty content is replaced by edema and microhemorrhages. Color-coded VNCa reconstructions show good to excellent results for qualitative assessment of BME, either in terms of sensitivity (range 72–96%), specificity (range 70–100%), and accuracy (range: 90–99%) (Table 2).

Similarly, receiver operating characteristic curve analysis of bone marrow attenuation on VNCa datasets has also demonstrated excellent sensitivity, specificity, and accuracy, ranging 85–96%, 82–90%, and 85–91%, respectively, using cutoff values from -80 and -12 HU [46, 56–58].

**Fig. 2:**
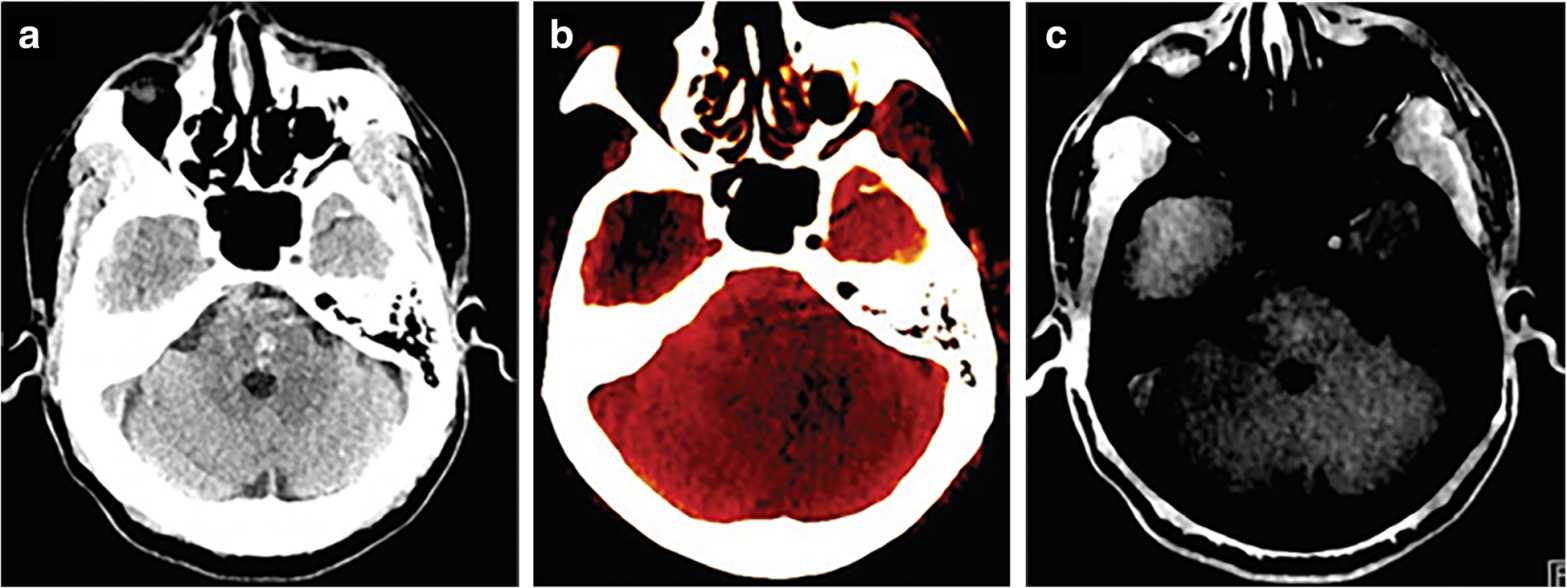
Head dual-energy computed tomography scan shows focal hyperattenuation in the pons, classified as indeterminate at standard grayscale series (**a**). This 58-year-old patient had a history of hypertension and diabetes and presented to the emergency department with right arm tremor, blurry vision, and increased systolic blood pressure. On the calcium-overlay image, the focal hyperattenuation in the pons was not apparent (**b**). On the virtual non-calcium image (**c**), the focal hyperattenuation in the pons manifests as a focal area of high attenuation, compatible with hemorrhage. From Hu et al. [17]

Interesting results have also been obtained for the diagnosis of sacral insufficiency fractures (Fig. 2), showing high sensitivity and specificity (93% and 95%, respectively) for qualitative assessment, and values of 85% and 95% for quantitative assessment using a cutoff value of -43 HU [29]. This may allow DECT to act as a promising technique to avoid misinterpretation of insufficiency fractures and their related complications, particularly in patients suffering from osteoporosis or diffuse bone disease [59].

#### Degenerative disc disease

Degenerative disease of intervertebral disc apparatus is a common age-related condition, causing lower back pain and entailing substantial social and economic burden [34].

Common complications are compressions of the spinal cord or spinal nerve root. Fast and accurate diagnosis is necessary for rapid initiation of optimal therapy and to avoid compressions that can result in irreversible morbidity. MRI is the preferred diagnostic imaging modality due to its ability to provide excellent demarcation between the intervertebral disc and cerebrospinal fluid [60]. However, MRI has several limitations in clinical routine, such as patients with ferromagnetic metallic implants, claustrophobia, or difficulties in staying supine and still for long acquisition times.

DECT imaging has been introduced as an attractive alternative, especially due to VNCa imaging, which achieved promising results for the identification of early stages of intervertebral disc degeneration. Using a maximum CT value of 800 HU and a threshold of -200 HU, VNCa color-coded maps with mixed CT overlay have demonstrated the ability to detect different grades of the modified Pfirrmann classification, which is widely used for disc degeneration grading [61]. In particular, VNCa imaging is able to detect an increase in disc attenuation that positively correlates to dehydration of nucleus pulposus and in a loss of its proteoglycan and water content.

**Fig. 3:**
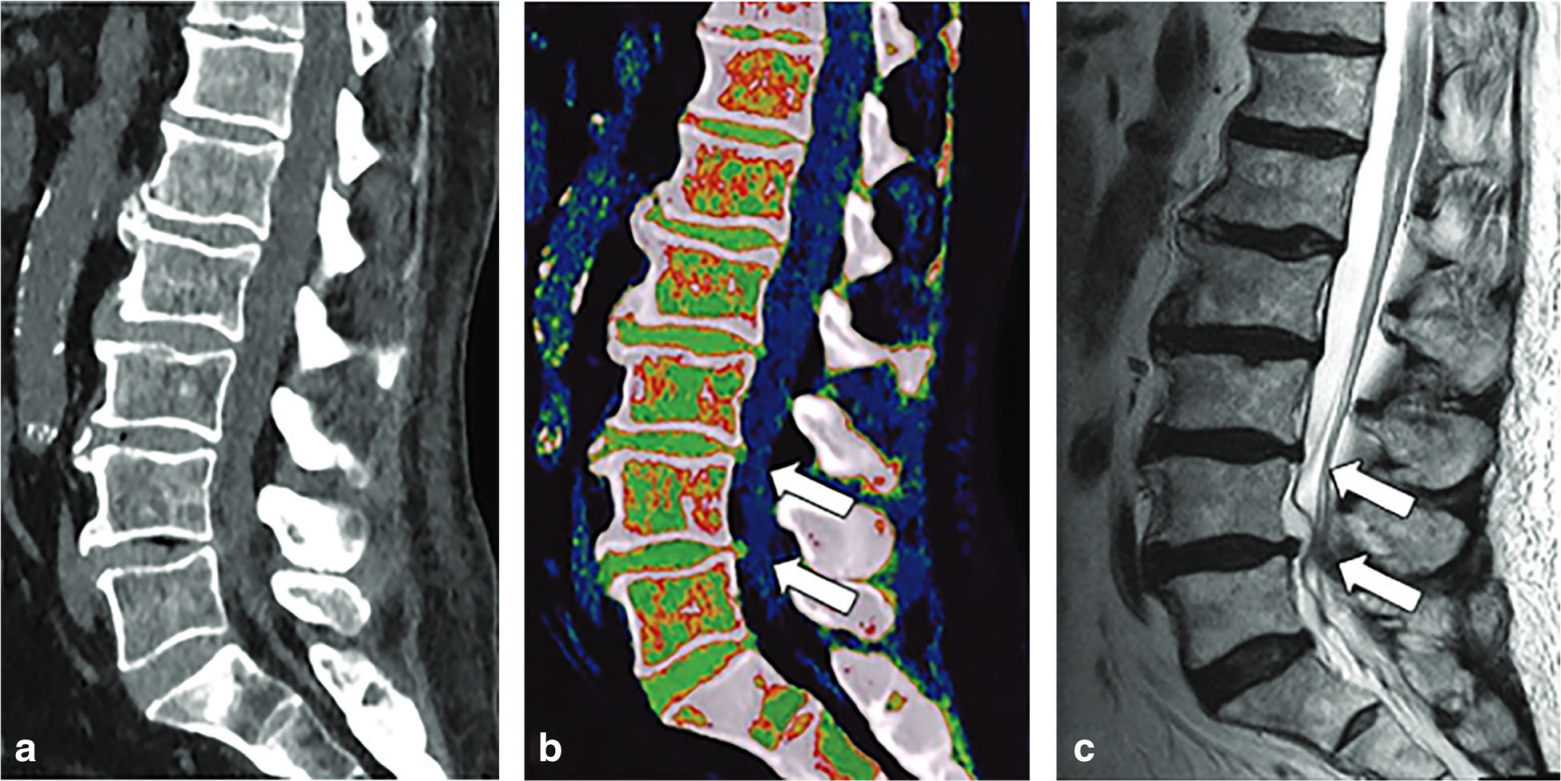
Spine dual-energy computed tomography. Standard grayscale series (**a**) shows typical findings of spondylarthrosis with vacuum phenomena in L3/L4 and L4/L5 intervertebral discs. Virtual non-calcium reconstruction with optimization for intervertebral disc analysis (**b**) can finely show the protrusion of lumbar discs (*arrows*), confirmed by magnetic resonance imaging T2-weighted sequence (**c**) (*arrows*). From Booz et al. [34]

More advanced stages of intervertebral disc degeneration are characterized by disc height reduction, fissuration of the annulus fibrosus, and herniation of the nucleus polposus. Standard CT has shown moderate sensitivity and specificity for the detection of lumbar disk herniation, despite the improved results coming from new iterative reconstruction algorithms [34, 62, 63]. More recently, DECT has overcome the impaired contrast resolution of intervertebral discs by application of color-coded VNCa reconstructions (Fig. 3).

Color-coded maps help distinguish small disc herniations from cerebrospinal fluid, with better sensitivity and specificity compared to standard CT, respectively of 91% and 92%, using MRI as a reference standard (Table 3) [34].

#### Infiltrative disease

The spine represents the most common site of bone metastases. Only breast, prostate and lung cancers are together responsible for more than 80% of cases of metastatic bone disease [64].

A contrast-enhanced CT scan is typically performed in oncologic patients either for staging and follow-up purposes [56]. However, despite iodine-based contrast agents helping enhance soft tissue contrast, the assessment of bone lesions on standard CT remains challenging. In a meta-analysis, conventional CT images reported a sensitivity and specificity of 77% and 83% for detection of spine metastasis [65]. Moreover, patients may need to undergo additional imaging techniques, such as MRI, scintigraphy, or positron emission tomography when there is high suspicion for bone metastasis presence [56].

The efficacy of VNCa reconstructions to detect metastatic spine lesions has been recently assessed with different calcium suppression indices. In particular, the use of low- and medium-suppression indices resulted in an increase in sensitivity to 85%, compared to 78% of conventional CT, and it was associated with a good inter-reader agreement at subjective image analysis [56].

In a study from Abdullayev et al. [56], quantitative analysis using low- and medium-suppression indices showed promising results to discriminate between normal and metastatic bone, using a cutoff of -143 HU and -31 HU, respectively (Table 3).

**Fig. 4:**
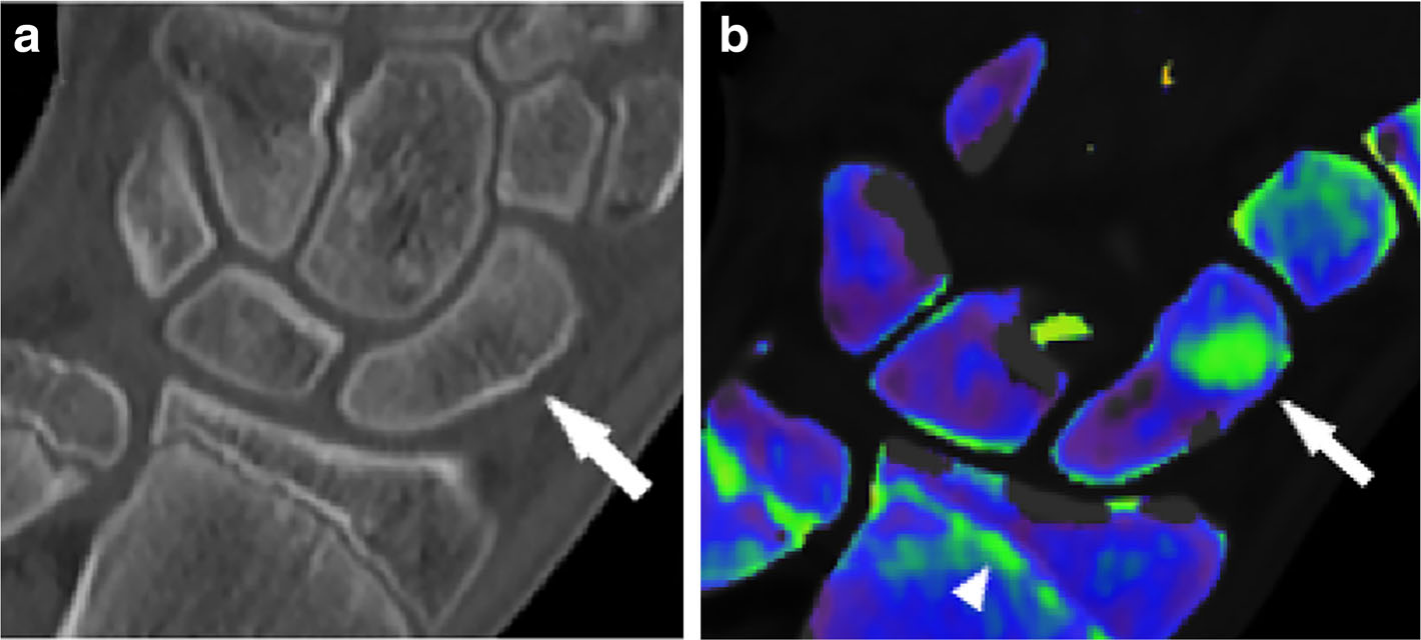
Hand dual-energy computed tomography scan in a patient with a non-displaced scaphoid fracture, which was confirmed at magnetic resonance imaging (not shown). Standard grayscale series (**a**) shows a subtle cortical interruption, which is not clearly suggestive for fracture (*arrow*). Color-coded virtual non-calcium image (**b**) depicts the presence of bone marrow edema confirming the hypothesis of a traumatic lesion. Of note, the epiphyseal line on the distal radius and ulna are also color-coded in green (*arrowhead*). From Dareez et al. [84]

The role of DECT in infiltrative spine disorders has also been focused on patients with multiple myeloma. In this malignant hematological tumor, the unbridled clonal proliferation of plasma cells causes an alteration of the normal components of bone marrow [66]. Low-dose total body CT scans are usually performed to detect osteolytic lesions [66]. In this setting, DECT has shown promising results, with better sensitivity compared to standard CT (Fig. 4).

Different studies have proposed threshold values ranging between -45 HU and -36 HU to achieve optimal sensitivity by quantitative assessment and to allow fine depiction either of focal and diffuse patterns of disease, with accuracy ranging from 93 to 99% [12, 66, 67].

### Appendicular skeleton

#### Hip

VNCa reconstructions are particularly helpful to detect subtle hip fractures that might be missed on conventional radiographs or standard CT, especially in patients affected by diffuse skeletal disorders such as osteoporosis or Paget’s disease [36].

Prompt identification of pelvic fractures is crucial for therapeutical planning and misdiagnosis is related to disability and higher mortality rates, with complications such as avascular necrosis and thromboembolism [68]. Different authors focused on the diagnostic performance of VNCa reconstructions to detect pelvic fractures, using clinical follow-up as a reference standard (Table 3). In these studies, DECT performed significantly better than standard CT, showing an improvement of sensitivity (> 5%) when color-coded VNCa images were evaluated [37, 69]. Moreover, quantitative analysis with a cutoff of -55.3 HU yielded sensitivity and specificity of 100% and 94%, respectively [36].

Different conditions such as axial spondylarthritis and sacroiliitis, involving either the spine or the pelvic girdle, usually require patients to undergo MRI. The VNCa algorithm has also been investigated for inflammatory changes of the bone marrow (Table 3), with results showing good diagnostic performance to highlight BME caused by active inflammation [70–74]. Promising results have also been shown for infiltrative lesions, and VNCa has been used as a guide for biopsy of malignant pelvic neoplasms that are barely visible on standard CT because of isodense bone marrow [75].

#### Limbs

A recent meta-analysis [76] compared the diagnostic performance of CT and MRI to establish a definitive diagnosis of a suspected fracture in small bones. MRI yielded superior sensitivity and specificity compared to CT (respectively 88% and 100% *versus* 72% and 99%), using bone scintigraphy as a reference standard. In case of suspected or subtle fractures, MRI is considered the best advanced imaging option after conventional radiography, as it finely depicts bone marrow and adjacent soft tissues. On the other hand, MRI alone may not depict fracture lines in case of intense BME, and conventional radiographs or CT may be needed for diagnosis [77]. Additionally, high cost, low access, and contraindications prevent MRI from playing a role as an advanced imaging option for suspected and occult fractures in emergency settings [76, 78]. However, bone bruise is considered the key finding of bone injury at MRI, as it allows uncovering subtle fractures even in small bones that might be occult at conventional radiography and standard CT [79].

An increasing number of studies have shown the feasibility of DECT to detect traumatic BME in small bones of appendicular skeleton exploiting VNCa imaging (Table 4), with improved diagnostic performance compared to standard CT either for qualitative and quantitative evaluation [80–83].

**Fig. 5:**
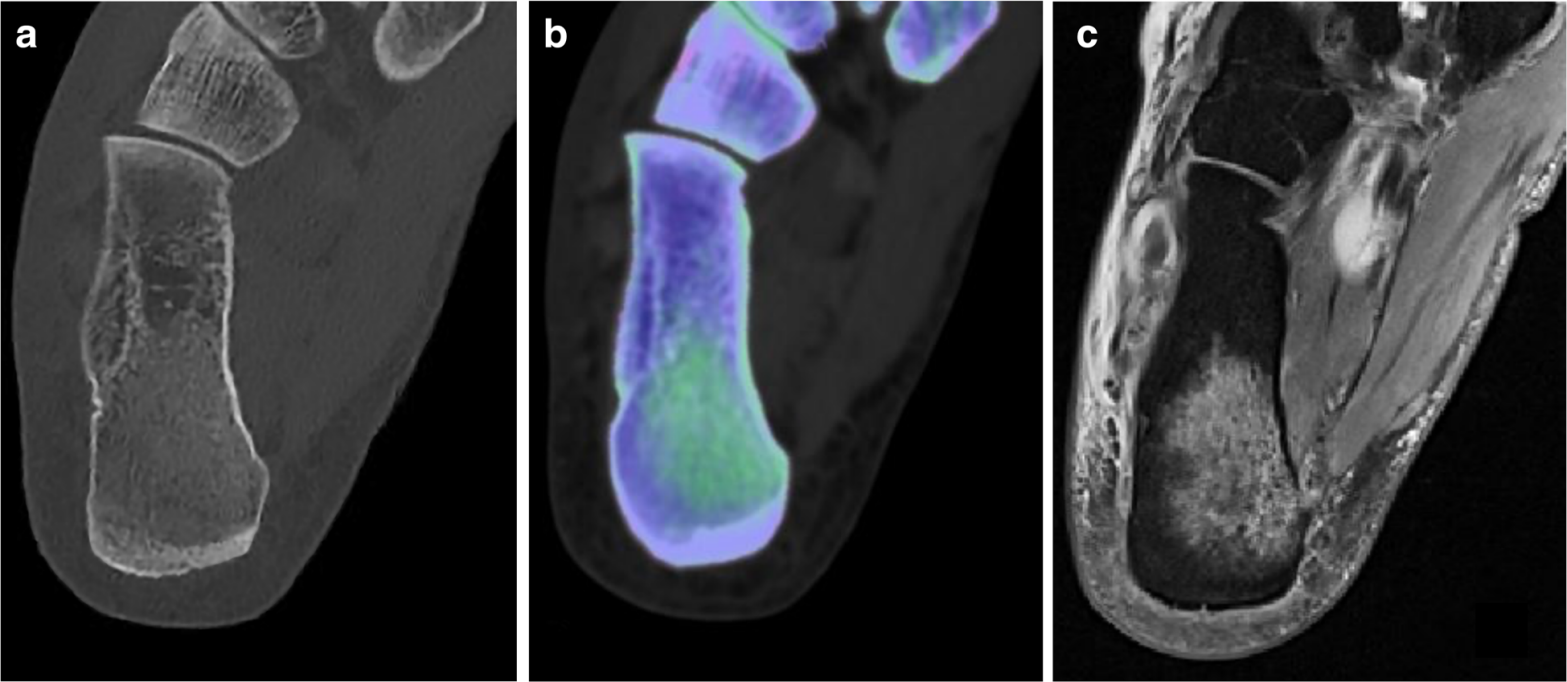
Dual-energy computed tomography scan in a patient presenting with right-sided acute ankle trauma. Standard grayscale series (**a**) does not depict any fracture line. Color-coded virtual non-calcium image (**b**) shows a distinct traumatic bone marrow edema of the right calcaneus, displayed as a green area (*arrow*). The finding was confirmed by magnetic resonance imaging (**c**) using a proton density-weighted sequence (*arrow*)

DECT can complement the information provided by standard CT imaging and enhance the diagnostic capabilities of VNCa for the evaluation of acute knee fractures (Fig. 5). In a study by Booz et al. [82], qualitative assessment of knee fractures by color-coded VNCa images yielded sensitivity and specificity of 95%, while at quantitative analysis, these values were 96% and 97%, respectively, using a -51 HU cutoff. Similar results have been shown by Wang et al. [80], who proposed a cutoff of -67 HU, yielding a sensitivity of 81% and specificity of 99%. Compared with standard CT, DECT has demonstrated a 15–20% increase in sensitivity to detect fractures, especially for less experienced radiologists [81]. The VNCa algorithm has been shown to perform well even with a low radiation-dose protocol, with excellent agreement with standard-dose DECT [48]. Bipartite patella and osteoarthritis can represent a pitfall of the VNCa algorithm, since they may determine false positives and negatives [81]. An additional limitation represents also the presence of prostheses or osteosynthesis implants [81].

Several authors have investigated the performance of VNCa reconstructions to detect traumatic BME in small bones of distal joints, such as the scaphoid [47, 84–88]. In these studies, DECT was able to highlight traumatic BME with higher sensitivity and specificity than standard CT either for qualitative assessment and quantitative analysis (Table 4) [85]. Some case reports also highlighted the ability of DECT to depict Achilles tendon tears with improved confidence over conventional CT [89]. Similar outcomes have been reported for evaluation of cruciate ligament rupture in acute knee trauma, with rather good sensitivity (79%) and excellent specificity (100%), using MRI as a reference standard [16].

**Fig. 6:**
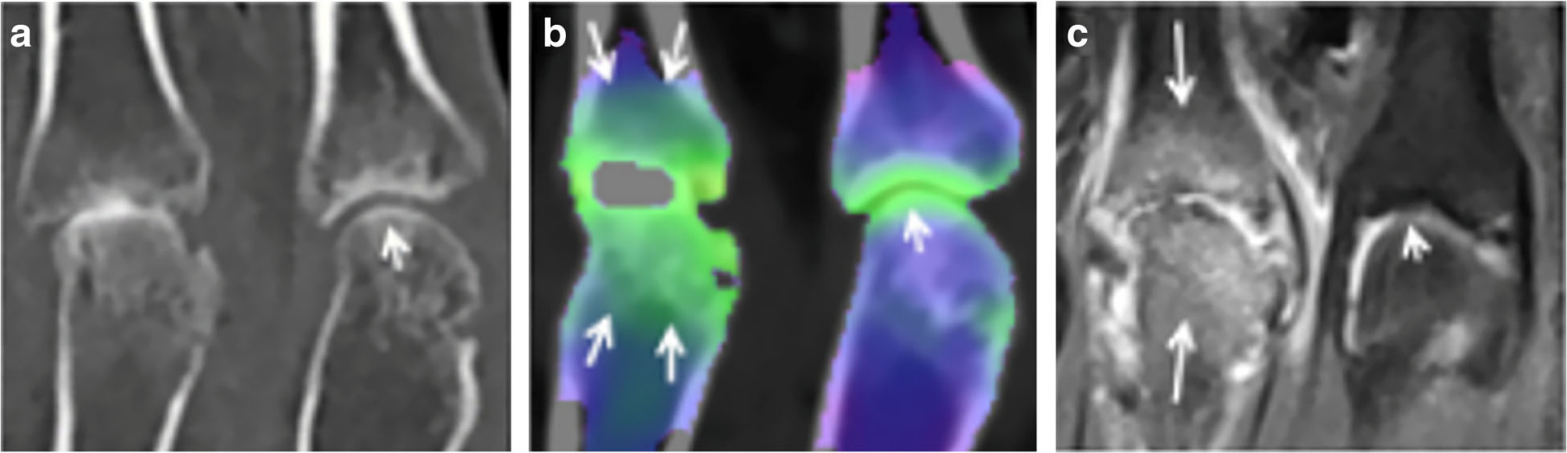
Dual-energy computed tomography scan of hands in a patient with rheumatoid arthritis. Magnification on the second metacarpophalangeal joint shows a normal and smooth outline of cartilage and bone plate (*short arrows*) on standard grayscale series (**a**) and normal virtual non-calcium attenuation (**b**). Conversely, bone marrow edema is evident on the third metacarpophalangeal joint presenting as an extensive and ill-defined green area (*long arrows*). Magnetic resonance imaging by means of T2-weighted fat-saturation sequence confirmed the presence of inflammatory bone marrow edema (**c**). From Jans et al. [72]

VNCa imaging performs well on small bones also to depict inflammatory BME, related to rheumatoid arthritis, in either large or small joints (Fig. 6), showing good qualitative assessment and excellent agreement with MRI [72].

## Vascular applications

Sufficient removal of calcified plaques from vessels represents a current challenge for CT angiography (CTA) studies. Calcified plaques commonly cause overestimation of vascular stenosis assessment, especially in small vessels, and may lead to unnecessary invasive procedures [90].

Usually, a fixed HU value is used as a threshold to remove calcifications in conventional CTA, despite this method often failing due to calcium blooming. Alternatively, unenhanced CT acquisition can serve as a mask for subtraction of calcified plaques. However, misregistration artifacts may occur due to patient’s movements or arterial pulsations between the two scans [13, 91]. DECT algorithms are well known to improve image quality, to reduce the contrast medium volume and the radiation dose to patients undergoing CTA studies [6, 13, 92–94]. DECT-based three-material decomposition also allows for subtraction of the calcium signal from iodinated vessels, permitting the removal of hard plaques from a CTA scan [95].

In this context, VNCa imaging has been shown to improve the quantification of carotid artery stenoses caused by hard plaques compared to conventional CTA, using digital subtraction angiography as reference [95]. Moreover, lumen assessment did not show to be impacted by blooming artifacts, probably because the algorithm recognizes and removes the spectral behavior of calcium blooming components [95].

## Future outlook

Energy-integrating detectors used in modern DECT platforms are based on an indirect process of x-ray conversion into an electrical signal, which passes by photodiodes and conversion into light photons [96]. On the other hand, new photon-counting detectors directly convert x-ray photons to an electrical signal, increasing dose efficiency and spatial resolution, and improving spectral separation together with its spatial registration. In the near future, photon-counting detector CT platforms may enable not only to visualize BME more in detail, especially in small bones, but also to differentiate among urate crystals, hydroxyapatite, and calcium pyrophosphate deposits [97]. Moreover, preliminary data have shown promising results for subtraction of calcified plaques from vessels, and separation between blood and brain calcifications, overcoming current VNCa limitations of suboptimal material decomposition and resolution restrictions of DECT platforms [98, 99].

However, we are still at the doorstep of this new era of CT technology and further investigation needs to be performed prior to introduction of photon-counting detector CT platforms into clinical routine.

## Conclusions

In the last ten years, DECT-based VNCa imaging has been shown to provide additional clinically relevant information compared to standard CT in several neuroradiology, vascular, and musculoskeletal applications. The greatest experience in using VNCa reconstructions exists in bone marrow imaging to date, particularly for trauma, but also for inflammatory and oncologic bone marrow pathologies. Several studies have demonstrated its potential also for the differentiation of hyperdense lesions as well as for intervertebral disc assessment. In addition, inflammatory or infiltrative bone marrow disorders that are conventionally assessed by means of MRI or bone scintigraphy can be assessed in more detail with DECT in comparison to standard CT. Subjective as well as objective analysis of VNCa images has shown high diagnostic accuracy and demonstrated its potential to serve as a viable imaging alternative to MRI, bone scintigraphy, or PET/CT in case of contraindications or limited availability. Especially in emergency/trauma settings, patients can substantially benefit from this technique due to time savings, early accurate diagnosis, and prompt therapy initiation. However, despite multiple studies having shown the potential of DECT-based VNCa imaging, this technique still has not gained ground in clinical routine, probably because of its limited availability considering that most of available studies have been performed on second- and third-generation DSCT platforms. This might have limited inter-vendor correlation studies and hampered the integration of VNCa imaging into clinical routine.

It is crucial to be aware of VNCa technical limitations and differences related to acquisition protocols and reconstruction software, which may differ across vendors, and generate different cutoff values for quantitative assessment. Further investigation of VNCa imaging algorithms is needed to gain a more comprehensive understanding of its potential.

## Data Availability

Not applicable

## Abbreviations

BME: Bone marrow edema
CTA: Computed tomography angiography
DECT: Dual-energy computed tomography
DE_ratio_: Dual-energy ratio
DSCT: Dual-source computed tomography
MRI: Magnetic resonance imaging
HU: Hounsfield units
PET: Positron emission tomography
VNCa: Virtual non-calcium
